# Expanding disease resistance: engineered NLRS for broad-spectrum protection

**DOI:** 10.1186/s43897-025-00217-4

**Published:** 2025-11-24

**Authors:** Tingting Zhou, Jiajun Wang, Yunjing Wang

**Affiliations:** School of Agriculture and Biology, Shanghai Jiao Tong University, Shanghai, 200240 China

﻿Plants constantly confront threats from microbial pathogens. To combat these challenges, they have evolved sophisticated defense mechanisms, most notably a dual‐layered immune system: pattern-triggered immunity (PTI) and effector-triggered immunity (ETI). PTI involves pattern recognition receptors (PRRs) detecting pathogen-associated molecular patterns (PAMPs) to activate broad-spectrum immunity (Boller and Felix 2009). Pathogens evade PTI via immunosuppressive effectors (Albert et al. 2020). This prompts plants to deploy ETI—a stronger, specific response mediated by NLRs (NB-LRR proteins) that recognize effectors (Bentham et al. 2017). Structurally, NLRs comprise three domains: N-terminal (CC or TIR), central NB-ARC, and C-terminal LRR, classifying them as CNLs/TNLs (Wang et al. 2021).

﻿The cultivation of crops carrying NLR genes remains the most effective strategy for disease control, particularly against viral pathogens. To date, 34 antiviral NLR genes such as *N*, *Rx1*, *Tm-2*^*2*^ and *Sw-5b* have been cloned from various plants for crop breeding and virus management (Zhu et al. 2025). However, NLR-mediated resistance faces several limitations. First, individual NLRs typically recognize only specific effectors, resulting in narrow-spectrum resistance. Second, while engineering approaches like site-directed mutagenesis and domain shuffling can expand recognition specificity, such "decoy engineering" strategies are constrained by the absence of compatible NLRs in many crop species (Kim et al. 2016).

To address these challenges and expand NLR specificity, a groundbreaking study from Liu lab recently proposed an innovative NLR engineering strategy for plant disease resistance breeding (Wang et al. 2025b).

Initially, Wang and colleagues noticed that CNL/RNL activity requires a free N-terminus, as N-terminal polypeptide tags inhibit their function. This strategy capitalizes on potyviral proteases, particularly NIa and its processed form NIa-Pro, which account for 30% of plant viruses (Wang et al. 2025b). These conserved cysteine proteases recognize specific cleavage motifs (xxVxxQ↓A(G/S) or xxVxHQ↓A(G/S)), providing ideal targets for engineering broad-spectrum resistance against potyviruses (Palani et al. 2021).

Wang et al. designed HA-PCS^PVY^-aTm-2^2^, a chimeric NLR containing the potato virus Y (PVY) NIa protease cleavage site fused to an autoactive Tm-2^2^ (aTm-2^2^) (Wang et al. 2025b). Co-expression of HA-PCS^PVY^-aTm-2^2^ and NIa-Pro^PVY^ by agrobacterium inoculation induced cell death, with PVY NIa (or NIa-Pro) cleavage confirmed by immunoblotting. They then generated transgenic *Nicotiana benthamiana* plants expressing HA-PCS^PVY^-aTm-2^2^. T1 transgenic plants showed normal growth but strong resistance to multiple potyviruses including PVY, Turnip mosaic virus (TuMV), pepper mottle virus (PepMoV), chilli veinal mottle virus (ChiVMV), and plum pox virus (PPV), demonstrating that the engineered CNL protein system confers wide-ranging protection through conserved protease recognition (Fig. 1).

**Fig. 1:**
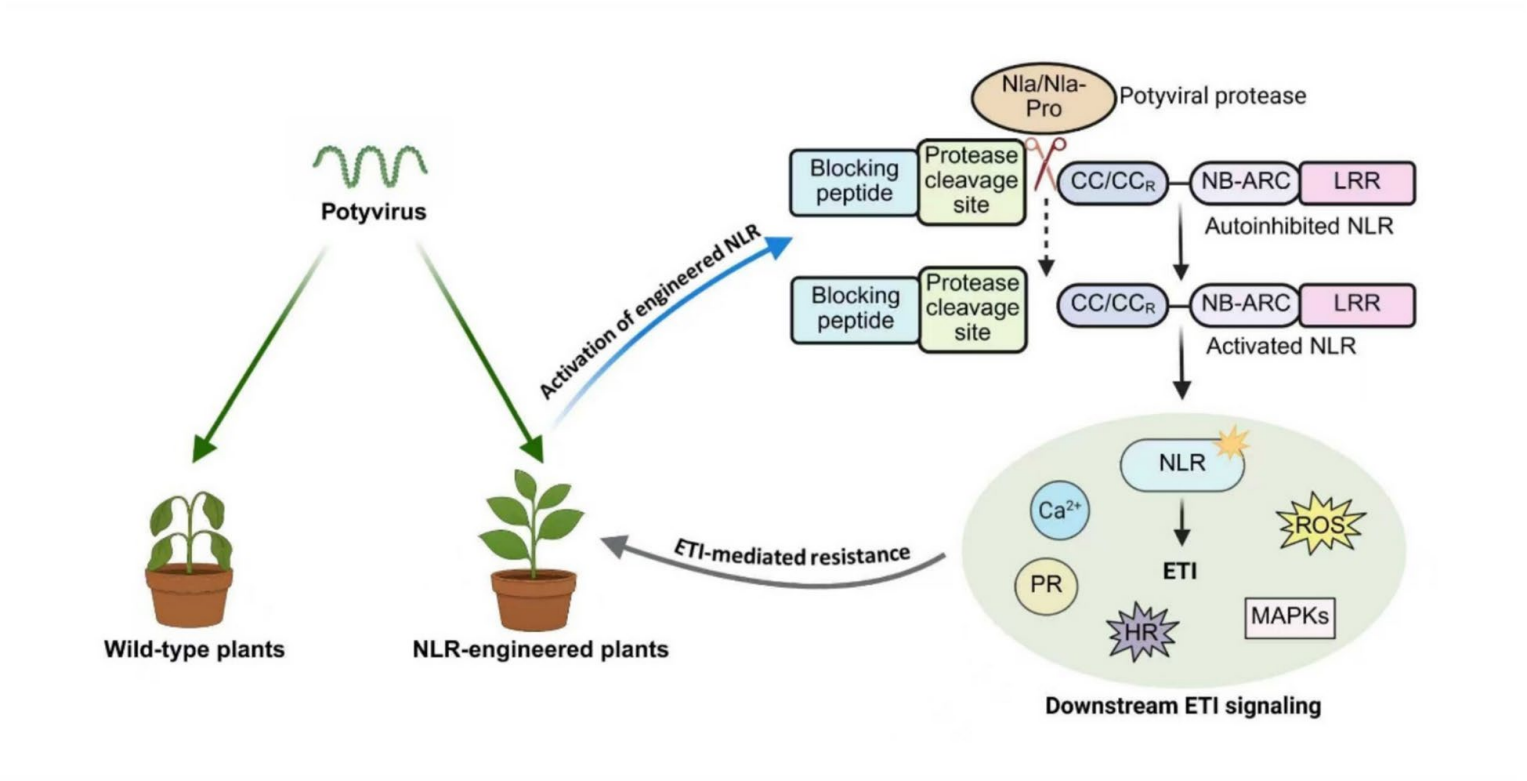
A simplified model for autoactive NLR-engineered plants conferring potyvirus resistance

Wang et al. then extended this strategy to RNLs, creating HA-PCS^PVY^-aAtNRG1.1 and HA-PCS^PVY^-aNbNRG1 variants (Wang et al. 2025b). Like their CNL counterpart, modified RNLs conferred complete resistance to PVY-GFP, TuMV, PPV, PepMoV and ChiVMV in T1 transgenic plants. These results establish that protease-sensing RNLs can similarly provide extreme resistance against multiple potyviruses.

Wang et al. also noticed that the original HA-PCS^PVY^-aNLRs (aTm-2^2^/aAtNRG1.1) failed to recognize tobacco etch virus (TEV) NIa, rendering plants vulnerable to TEV infection (Wang et al. 2025b). To address this limitation, they developed a tandem cleavage site (HA-PCS^TEV^-PCS^PVY^-aAtNRG1.1), targeting both PVY and TEV proteases. This dual-recognition system successfully induced complete resistance to PVY, TuMV, PPV, PepMoV, and ChiVMV; and partial-to-complete TEV resistance. Finally, Wang et al.explored the potential of redesigning aNLR in crops protection (Wang et al. 2025b). They selected soybean mosaic virus (SMV) and soybean as research models. By engineering HA-PCS^SMV^-aAtNRG1.1 with the SMV-specific cleavage site, they demonstrated complete SMV resistance in transgenic soybean plants. This successful translation to an important crop validates the protease-cleavable NLR strategy as a viable approach for developing high-yielding, virus-resistant cultivars.

## Future perspectives

Current NLR engineering strategies primarily involve modifying sensor NLRs to alter effector recognition profiles, as exemplified by decoy engineering and domain shuffling. However, these approaches are often limited by the rapid evolution of plant pathogens and may lack broad-spectrum efficacy and durability under field conditions. For instance, the NLR RPS5 is activated when its decoy protein PBS1 is cleaved by a pathogen-secreted protease (Wang et al. 2025a). Although this system broadens NLR specificity, engineering such decoys remains technically challenging and is effective only in plant species that possess PBS1-guarding NLRs (Wang et al. 2025a). Another domain shuffling example comes from the rice NLR pair Pik-1/Pik-2, which confers resistance to blast fungus. Replacing the integrated HMA domain of Pik-1 with nanobodies against GFP or mCherry represents an innovative reprogramming of recognition; however, obtaining functional nanobodies is often difficult and time-consuming (Wang et al. 2025a). In contrast, Wang et al. developed a novel NLR engineering strategy that diverges from conventional methods (Fig. 1) (Wang et al. 2025b). Using a simple and rapid process to generate chimeric proteins, their system confers complete resistance against at least 110 species of all known potyviruses, with even broader coverage achieved through tandem cleavage sites. This approach offers the following key advantages:

### Simplicity and generality

The design requires only a single engineered autoactive NLR, eliminating the need for decoy proteins. It relies on pathogen protease-mediated cleavage at introduced sites rather than specific effector recognition, thereby avoiding time-consuming screens for effective decoys or R-Avr combinations.

### Compatibility with genome editing

The system is well-suited for integration with advanced genome editing technologies. For example, base editing could introduce point mutations to generate constitutively active NLRs, and gene knock-in techniques could insert short sequences encoding cleavage site-containing polypeptides into crop genomes to enhance immunity.

### Broad-spectrum potential

A single aNLR can target conserved protease cleavage sites across multiple pathogens and crop species. Systematic exploration of conserved protease motifs and analogous pathogen-derived signals beyond potyviruses could help establish universal activation codes for NLR-mediated immunity.

### Durability

Resistance conferred by this strategy is expected to be durable, since loss of protease function in the pathogen would likely disrupt essential protein processing, resulting in lethal effects or significant fitness costs.

Therefore, this approach represents a "paradigm shift" in plant immunity engineering:

1. The cross-species functionality of this system may arise from the conservation of downstream signaling components. Upon proteolytic activation, the aNLR is hypothesized to generate a generic “cell death signal” that can be recognized and amplified by conserved downstream pathways across different species. Supporting this notion, Du et al. successfully bypassed taxonomic constraints by co-expressing Solanaceae sensor NLRs with their cognate NRC helper NLRs in rice, soybean, and Arabidopsis, demonstrating that immune functionality can be transferred across evolutionarily divergent species (Du et al. 2025). These findings raise the intriguing possibility of designing broad-spectrum resistance elements that could be deployed universally across multiple crops. Nevertheless, the application of such an aNLR strategy does not preclude the need for species-specific NLR engineering, and the cross-species functionality of this approach requires systematic validation in each target crop.
2. This strategy represents a paradigm shift from strain-specific to broad-spectrum and durable resistance. By targeting functionally conserved proteases secreted by diverse pathogens including fungi, oomycetes, bacteria, and viruses, a single protease-activated NLR system can theoretically provide resistance across multiple species. The main challenge is ensuring the targeted protease active site region is conserved enough for a single aNLR design, while also being sufficiently divergent from plant endogenous proteases to avoid off-target cleavage and autoimmunity. Besides, in agricultural settings, crops are frequently co-infected by multiple pathogens. However, this system is ineffective against pathogens that lack PCSs. Consequently, the protective function may be impaired, and off-target effects could arise.
3. This strategy marks a paradigm shift from the traditional "gene-for-gene" approach, to targeting core virulence functions. The core concept of hijacking pathogen activity to trigger defense is not limited to proteases. This modular design could extend to other pathogen enzymes that cleave or modify proteins. For example, an aNLR could be engineered with an activation domain masked by a ubiquitination tag. Upon detection of bacterial E3 ubiquitin ligases, the tag could be removed, activating immune responses.

As for implementation, this innovative strategy could be integrated with CRISPR–Cas genome editing to modify endogenous *R* genes and conditionally activate endogenous NLRs, thereby enhancing plant immunity. Besides, combining artificial intelligence-based computational protein design and structure prediction methods may facilitate the exploration of conserved protease cleavage motifs across diverse pathogen taxa beyond potyviruses, and systematic exploration of conserved protease cleavage motifs and analogous pathogen-derived signatures could help define universal activation codes for NLR-mediated immunity.

Autoactive NLRs were engineered by incorporating a viral protease cleavage site. CNL/RNL activity requires a free N-terminus, as N-terminal polypeptide tags inhibit their function, hence incorporating a protease-cleavable tag at the N-terminus of autoactive NLRs could create pathogen-responsive immune switches. Potyviruses could generate NIa or NIa-Pro proteases. Upon cleavage by potyviral proteases, the modified NLRs activate, triggering immune responses that confer resistance to viral infection in plants. The green arrow denotes potyvirus infection, the blue arrow represents the activation framework in aNLR-engineered plants, and the grey arrow indicates the activated effector-triggered immunity response in these plants. Abbreviations: PR, pathogenesis-related genes; HR, hypersensitive response; ROS, reactive oxygen species; MAPKs, mitogen-activated protein kinases. Created with BioRender.com.

## Data Availability

Not applicable.
